# [Retracted] Promoter methylation of *RASSF1A* modulates the effect of the microtubule-targeting agent docetaxel in breast cancer

**DOI:** 10.3892/ijo.2025.5745

**Published:** 2025-04-14

**Authors:** Eun Young Gil, Uk Hyun Jo, Hoiseon Jeong, Young Mi Whang, Ok Hee Woo, Kyu Ran Cho, Jae Hong Seo, Aeree Kim, Eun Sook Lee, Insong Koh, Yeul Hong Kim, Kyong Hwa Park

Int J Oncol 41: 611-620, 2012; DOI: 10.3892/ijo.2012.1470

Following the publication of this paper, and a corrigendum that was subsequently published to take account of anomalies that were identified with the assembly of western blot data in Fig. 5B (doi: 10.3892/ijo.2017.3900), a concerned reader drew to the Editor's attention that, regrettably, there remained possible issues with the duplication of data in the revised version of Fig. 5 provided by the authors in the Corrigendum; in addition, potential anomalies were also noted with the western blot assay data in the originally published versions of Fig. 2A and B.

After having performed an independent review of the data in these figures in the Editorial Office, the concerns of the reader were found to be validated. Therefore, the Editor of *International Journal of Oncology* has decided that this paper should now be retracted from the Journal. After contacting the authors, they accepted the decision to retract the paper. The Editor apologizes to the readership for any inconvenience caused.

